# Over-Expression of TNFRSF12A Promotes Immune Suppression and Facilitates Angiogenesis in Triple-Negative Breast Cancer

**DOI:** 10.3390/biology14111513

**Published:** 2025-10-28

**Authors:** Can Jiang, Zhengwei Zhou, Guang Shu, Gang Yin, Maonan Wang

**Affiliations:** Department of Pathology, Xiangya School of Basic Medical Sciences, Central South University, Changsha 410013, China; 246511075@csu.edu.cn (C.J.); zhouzw@csu.edu.cn (Z.Z.); shuguang78@csu.edu.cn (G.S.); gangyin@csu.edu.cn (G.Y.)

**Keywords:** tumor immune microenvironment, triple-negative breast cancer, TNFRSF12A, angiogenesis

## Abstract

**Simple Summary:**

Triple-negative breast cancer (TNBC) is the most lethal of the breast cancer subtypes, exhibiting high levels of aggressiveness, recurrence, and metastasis. The absence of effective TNBC-specific biomarkers contributes to inadequate treatment responses and unfavorable patient outcomes, emphasizing an urgent necessity for novel therapeutic targets. This study, which analyzed 442 samples, identified TNFRSF12A as being highly expressed in TNBC. High TNFRSF12A expression has been demonstrated to be associated with unfavorable prognoses in clinical samples, a correlation that has been validated in clinical studies. This is the first report of TNFRSF12A overexpression in TNBC, suggesting its potential as an independent prognostic or diagnostic biomarker. A series of empirical investigations, encompassing single-cell analysis, T-cell cytotoxicity assays, and angiogenesis assays, have collectively substantiated that TNFRSF12A orchestrates a comprehensive remodeling of the immune microenvironment and a pronounced promotion of angiogenesis, thus propelling the progression of TNBC. This work provides the first cellular-level validation of TNFRSF12A’s functional role in TNBC, highlighting its significance for further mechanistic exploration and targeted drug development.

**Abstract:**

The urgent need for novel therapeutic strategies in triple-negative breast cancer (TNBC)—characterized by absent ER, PR, and HER2 expression—stems from its association with a paucity of effective treatments and an adverse prognosis. This study identifies TNFRSF12A as a key gene specifically overexpressed in TNBC versus other subtypes. Validation with clinical specimens confirmed its exclusive upregulation in TNBC tissues, correlating significantly with worse patient outcomes. Functional enrichment analysis (STRING/DAVID) indicated TNFRSF12A’s primary involvement in pathways positively regulating cell migration, angiogenesis, and hypoxia response. Immune infiltration profiling (TIMER/TISCH2) revealed selective enrichment of TNFRSF12A in cancer-associated fibroblasts (CAFs). Its expression showed a significant positive correlation with the CAF marker FAP (ρ = 0.304) and CAF infiltration levels, but inverse correlations with CD8^+^ T-cell (Cor = −0.165) and B-cell (Cor = −0.164) infiltration. Regarding chemoresistance, elevated TNFRSF12A expression significantly increased sensitivity to docetaxel. Molecular docking simulations further verified direct binding between TNFRSF12A and docetaxel, mediated by hydrophobic interactions and hydrogen bonds. To elucidate the underlying molecular mechanisms, cellular experiments revealed that TNFRSF12A knockdown resulted in (1) significantly compromised angiogenic capacity in HUVEC tube formation assays (*p* < 0.01); (2) markedly augmented cytotoxicity of T cells against tumor cells (*p* < 0.05); and (3) reduced cellular sensitivity to docetaxel, as evidenced by significantly elevated IC_50_ values in CCK-8 assays (*p* < 0.01). In summary, this study systematically elucidates how TNFRSF12A propels TNBC malignant progression by remodeling the tumor immune microenvironment and promoting angiogenesis. Concurrently, we reveal a TNFRSF12A-mediated chemosensitizing effect towards docetaxel. Therefore, these results are crucial for improving the targeting of TNFRSF12A and developing precise combination treatment regimens to improve outcomes for patients with TNBC.

## 1. Introduction

Globally, triple-negative breast cancer (TNBC) imposes a substantial health burden and ranks among the primary causes of cancer death in women due to its aggressive clinical behavior, heightened propensity for local recurrence, and elevated rates of distant metastasis [1]. A key defining feature of TNBC, setting it apart from other breast cancer subtypes, is the lack of expression of three fundamental receptors [2,3,4]. Currently, the lack of robust TNBC-specific diagnostic and prognostic biomarkers contributes to suboptimal responses to conventional therapies, universally poor clinical outcomes, and eventual tumor recurrence or metastasis in the majority of patients [5,6,7,8]. Consequently, there is a critical need to identify novel therapeutic targets for TNBC to improve patient prognosis.

While the role of the immune system in tumorigenesis and progression has been extensively documented, immunotherapy has only recently emerged as a transformative breakthrough in oncology, demonstrating remarkable antitumor efficacy and the potential for durable remission or cure [9]. Preclinical studies demonstrate compelling evidence of a significant synergistic interaction between chemotherapeutic agents and immune system functionality in TNBC, thus cooperatively potentiating anti-tumor immune responses [10,11,12]. The expression levels of particular immunological markers also show significant predictive value for chemotherapy benefit magnitude in TNBC [13,14]. However, the immunomodulatory mechanisms underlying specific therapeutic agents and their clinical translational potential remain incompletely characterized, warranting further investigation [15,16]. By delineating the molecular interplay of pivotal immune-associated genes in the immunologically suppressed niche of TNBC, this study endeavors to characterize immune-regulatory mechanisms operative therein. thereby providing a theoretical foundation for developing precision-based therapeutic approaches targeting the TME.

Tumor necrosis factor receptor superfamily member 12A (TNFRSF12A; commonly known as Fn14) constitutes a prominent constituent within the tumor necrosis factor receptor superfamily (TNFRSF) [17,18]. As a type I transmembrane glycoprotein, TNFRSF12A structurally harbors characteristic cysteine-rich domains (CRDs). TWEAK/TNFSF12 binding to the Fn14 ectodomain activates complex downstream signaling cascades [18]. The protein encoded by this gene exerts crucial functions across diverse physiological and pathological processes; it holds a central position, particularly in tissue damage repair, inflammatory responses, and the regulation of cellular homeostasis [19,20]. Accumulating research has progressively unveiled the pathogenic mechanisms of TNFRSF12A in diverse diseases, particularly malignancies and fibroinflammatory disorders [21,22,23,24], establishing it as a promising therapeutic target for disease diagnosis and intervention.

Leveraging breast cancer datasets from the GEO database, this study identified TNFRSF12A as a pivotal research target through differential gene expression analysis and Venn intersection screening with immune-related gene sets. Analytical findings revealed significantly elevated TNFRSF12A expression in TNBC tissues, positively correlated with detrimental clinical outcomes—shortened overall survival (OS; HR = 1.89, (1.28–2.81), *p* = 0.0012), reduced recurrence-free survival (RFS; HR = 1.47, (1.17–1.84), *p* = 0.00077), and worsened distant metastasis-free survival (DMFS; HR = 1.86, (1.35–2.57), *p* = 0.00011). Gene set enrichment analysis implicated this gene in tumor-associated neovascularization regulation, with experimental validation demonstrating markedly impaired vascular formation capacity following TNFRSF12A knockdown. Further immune infiltration assessment established an inverse correlation between heightened TNFRSF12A expression levels and infiltration levels of CD8^+^ T cells and B lymphocytes. In vitro cytotoxicity assays revealed that genetic ablation of TNFRSF12A augmented T-cell-mediated tumor cell lysis. Single-cell transcriptomic profiling revealed selective enrichment of TNFRSF12A expression in cancer-associated fibroblasts (CAFs), with its expression levels positively correlating with fibroblast infiltration density in the tumor microenvironment (McCluster algorithm: Cor = 0.316, *p* = 2.15 × 10^−5^; TIDE algorithm: Cor = 0.329, *p* = 9.12 × 10^−6^). Pharmacological susceptibility analysis coupled with experimental validation confirmed significantly increased sensitivity to docetaxel in high TNFRSF12A-expressing cohorts (validated by CCK-8 assay, *p* < 0.01). Molecular docking simulations identified potential binding sites between TNFRSF12A protein and docetaxel (binding energy < −6.7 kcal/mol). Collectively, these findings establish TNFRSF12A as a promising therapeutic target for TNBC with clinical potential for docetaxel combination regimens.

## 2. Materials and Methods

### 2.1. Computational Breast Cancer Data Analysis

R (Version 4.4.1) provides a freely accessible computational environment for statistical analysis, data visualization, and data mining. We employed the limma package (version 4.3) within this ecosystem to curate and analyze breast cancer datasets sourced from GEO repositories.

### 2.2. Patient Tissue Samples

Paired tumor and adjacent normal tissues from nineteen treatment-naïve triple-negative breast cancer (TNBC) patients were procured from Xiangya Hospital, Central South University (Changsha, China). All specimens underwent formalin-fixed paraffin-embedding (FFPE) without prior exposure to chemotherapy, radiotherapy, or immunotherapy. This study protocol received ethics approval from the Institutional Review Board of Xiangya Hospital.

### 2.3. Isolation of RNA from Tissue Samples

Following dewaxing with xylene, total RNA was extracted from formalin-fixed paraffin-embedded (FFPE) triple-negative breast cancer and normal tissue specimens using the AmoyDx^®^ FFPE RNA Isolation Kit (AmoyDx, Xiamen, China) per manufacturer’s protocols.

### 2.4. Gene Expression Analysis

UALCAN (https://ualcan.path.uab.edu/ (accessed on 22 October 2025)) is an interactive web portal enabling multi-omics analytical capabilities leveraging The Cancer Genome Atlas (TCGA) database. Its functionalities encompass transcriptomic differential expression profiling, survival outcome assessment, correlation analysis, protein-level expression comparisons, and non-coding RNA (miRNA/lncRNA) investigations. The platform further facilitates visualization of analytical outputs derived from TCGA oncogenomic datasets. We employed this resource to interrogate TNFRSF12A expression patterns in triple-negative breast carcinoma while concurrently retrieving genes exhibiting expression correlation with TNFRSF12A across breast cancer subtypes.

The HPA constitutes (https://www.proteinatlas.org) a comprehensive proteomic knowledgebase dedicated to exhaustive characterization of human gene and protein expression patterns. This resource integrates immunohistochemistry (IHC), immunofluorescence, and high-throughput antibody generation technologies, encompassing spatial distribution data for >24,000 human proteins across normal tissues, neoplastic specimens, cell lines, and hematopoietic cells. Leveraging the platform’s IHC repository, we extracted expression profiles of TNFRSF12A in breast carcinoma tissues relative to normal counterparts.

### 2.5. Prognostic Analysis

Employing the KM Plotter platform (https://kmplot.com/analysis/ (accessed on 22 October 2025))—an established online resource for survival analysis integrating data from GEO and TCGA repositories—we examined the prognostic impact of TNFRSF12A in triple-negative breast cancer patients. This comprehensive repository enables biomarker discovery and validation by establishing associations between gene expression and survival outcomes across >30,000 samples encompassing 21 malignancies.

### 2.6. Exploring the Function of TNFRSR12A

STRING (https://string-db.org/) constitutes a protein–protein interaction (PPI) network resource integrating evidence from public repositories and curated literature. It aggregates computationally processed data from multiple primary sources—including UniProt, KEGG, NCBI, and Gene Ontology—to generate a unified PPI network framework. We leveraged this platform to delineate putative interactors of TNFRSF12A and performed functional enrichment profiling of these candidate binding partners using its analytical framework.

The DAVID bioinformatics resource (https://davidbioinformatics.nih.gov/) (accessed on 22 October 2025) provides an integrated analytical platform that synthesizes biological data and computational tools. This system delivers comprehensive functional annotation for extensive gene/protein inventories (spanning hundreds to thousands of identifiers), enabling extraction of actionable biological insights from omics-scale datasets. Leveraging DAVID’s analytical infrastructure, we conducted functional annotation profiling of genes exhibiting expression correlation with TNFRSF12A.

The BioLadder platform (https://www.bioladder.cn/web/ (accessed on 22 October 2025) ) provides an integrated bioinformatics environment for computational analysis and analytical visualization. Utilizing this resource, we visualized enrichment analysis outputs, specifically prioritizing the top-ranked statistically significant enrichment terms (*p* < 0.05) for graphical representation.

### 2.7. Tumor Microenvironment Immune Cell Profiling

The TIMER platform (https://cistrome.shinyapps.io/timer/ (accessed on 22 October 2025)) serves as a dedicated computational resource for deconvoluting immune cell infiltration landscapes within tumor microenvironments. Leveraging TCGA RNA-Seq datasets, it quantifies abundance profiles of six major immune lineages. We employed this analytical framework to delineate TNFRSF12A-mediated modulation of immune infiltration dynamics in TNBC.

### 2.8. Single-Cell Data Analysis

The TISCH2 database (http://tisch.comp-genomics.org (accessed on 22 October 2025)) curates single-cell transcriptomic data encompassing 6,297,320 cells derived from 190 distinct datasets. Leveraging this resource, we deciphered the transcriptomic landscape across individual cells within breast carcinoma tissues. This analytical approach enabled comprehensive mapping of TNFRSF12A expression profiles among diverse cellular subtypes and subpopulations, thereby elucidating its spatial distribution patterns and functional relevance within the tumor ecosystem.

### 2.9. Drug Sensitivity Analyses

GSCA (https://guolab.wchscu.cn/GSCA/ (accessed on 22 October 2025)) represents an integrative computational platform for comprehensive oncogenomic investigations, consolidating functionalities spanning single-gene profiling, multi-gene signature analysis, immune infiltration quantification, mutational landscape assessment, and pharmacological responsiveness evaluation. This repository encompasses data from TCGA and Genomics of Drug Sensitivity in Cancer (GDSC) across 33 malignancy types. Utilizing its drug sensitivity module, we systematically assessed correlations between TNFRSF12A expression levels and chemotherapeutic responsiveness.

### 2.10. Molecular Docking Simulation of TNFRSF12A and Docetaxel

The PubChem database can be utilized to identify the three-dimensional structure of docetaxel, while the tertiary structure of TNFRSF12A was acquired from PDB. Molecular preprocessing of both ligand (docetaxel) and receptor (TNFRSF12A), including binding site specification, was performed using AutoDockTools (v1.5.7). Subsequent molecular docking simulations employed AutoDock Vina (v1.2.3) utilizing its integrated Iterated Local Search global optimization algorithm to identify optimal binding conformations. Resultant docking complexes underwent structural analysis and visualization primarily through PyMOL molecular graphics software (Version 3.0.3).

### 2.11. The Culture of Triple-Negative Breast Cancer Cell Lines

MDA-MB-231 and HCC1806 (the American Type Culture Collection, ATCC, Manassas, VA, USA) were utilized. For the cultivation of MDA-MB-231 cells, we utilized high-glucose DMEM medium. This medium contained 10% FBS and 1% penicillin–streptomycin, while HCC1806 cells were grown in RPMI 1640 containing identical concentrations of FBS and antibiotic/antimycotic. All cultures were maintained at 37 °C in a humidified 5% CO_2_ incubator, with routine testing confirming mycoplasma-, bacterial-, and fungal-free status.

### 2.12. Cell Transfection

All small interfering RNAs (siRNAs) utilized in this investigation were synthesized by RiboBio (Guangzhou, China). We seeded cells in logarithmic growth phase into 6-well plates. Upon reaching 70–90% confluence, transfection was conducted using Lipofectamine 3000^®^ (Invitrogen, Waltham, MA, USA), with all procedures adhering strictly to manufacturer protocols. Nucleotide sequences of experimental siRNAs are detailed in Table 1.

### 2.13. Real-Time Fluorescent Quantitative Polymerase Chain Reaction

The RNA was extracted using the RNA Isolater reagent (Vazyme, Nanjing, China). The extracted RNA was converted into cDNA using reverse transcription reagent (TransGen Biotech, Beijing, China). Then, the cDNA was analyzed by quantitative polymerase chain reaction (qPCR) according to a standardized protocol. We used the 2^−ΔΔCt^ method to calculate the expression levels of the target genes. The primer sequences listed in Table 2 are primers designed for the gene in this study.

### 2.14. In Vitro T-Cell Cytotoxicity Assay

Post-transfection, tumor cells were subsequently cultivated in a 12-well plate (1 × 10^6^ cells) and incubated for 24 h under standard conditions. Activated peripheral blood mononuclear cells (PBMCs) were then introduced at a 1:5 effector-to-target ratio (tumor cells: PBMCs) for 48-h co-culture. After 48 h of co-culture, the old culture medium was discarded. Then, the cells were fixed at room temperature with 4% paraformaldehyde for 30 min. Finally, staining with 0.1% crystal violet was performed to assess tumor cell death.

### 2.15. Tube Formation Assay

HUVEC cells were cultured in a 48-well plate (1.6 × 10^4^ cells per well) and cultured using conditioned medium collected under different treatment conditions. After an 8-h incubation period under standard conditions (37 °C, 5% CO_2_), images of endothelial tubulogenesis were acquired using light microscopy. The resulting tubular networks were quantitatively assessed utilizing the Angiogenesis Analyzer toolset within Image J software (Version 1.54).

### 2.16. CCK-8 Cytotoxicity Assay

At 24 h post-transfection, cells were plated in 96-well plates (5 × 10^3^ cells/well) with triplicate wells per group and cultured for 24 h at 37 °C. Docetaxel was then administered at graded concentrations. After a 48-h drug exposure, 10 μL of CCK-8 reagent was added per well, with incubation proceeding for 2 h. Absorbance (OD_450_) was measured using a microplate reader. For cell viability, the IC_50_ was derived from dose–response relationships.

### 2.17. Western Blot Analysis

Total protein was extracted from cell samples using RIPA lysis buffer (Beyotime, Shanghai, China) supplemented with a protease inhibitor cocktail. Total proteins were separated by SDS-PAGE and transferred to PVDF membranes (Cytiva, Marlborough, MA, USA) for detection. Subsequently, membranes were blocked in TBST solution containing 5% skim milk for 2 h, incubated with primary antibody overnight at 4 °C, and incubated with secondary antibody for 1 h at room temperature. Final visualization was achieved using chemiluminescent substrate (Biosharp, Beijing, China). The specific primary antibodies were as follows: GAPDH (Utibody; UM4002, 1:3000), VEGFA (LITHO; LTO0784, 1:3000).

### 2.18. Statistical Analysis

The GraphPad Prism 9.0 software was utilized to execute all statistical analyses in this study. Unpaired Student’s *t*-tests were employed for intergroup comparisons and assessments against control groups. For qPCR analyses of patient-derived samples, paired Student’s *t*-tests were applied. All experiments comprised ≥3 independent biological replicates. *p* ≤ 0.05 is considered to be statistically significant.

## 3. Results and Discussion

### 3.1. Identify TNFRSF12A for Subsequent Studies Based on Bioinformatics Analysis

**Figure 1:**
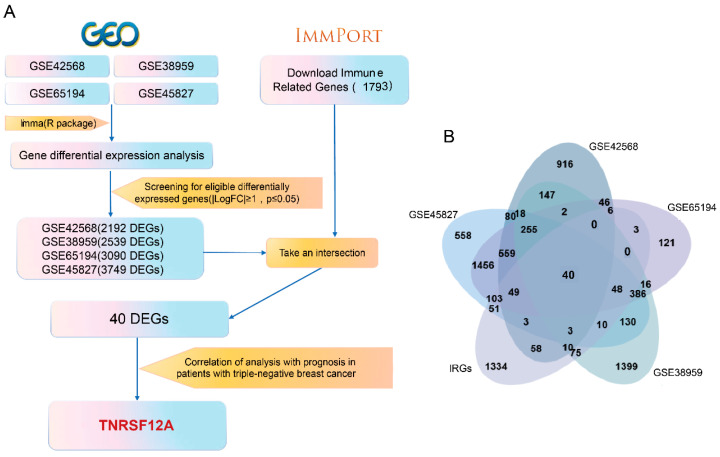
Identification Workflow for Key Immune-Related Genes Impacting TNBC Prognosis. (**A**) Schematic of the screening methodology. (**B**) Intersection of DEGs across four datasets with immune-related gene sets.

Publicly available breast cancer gene expression datasets were acquired from the GEO database: GSE69194 (153 breast cancer and 11 normal samples), GSE38959 (30 breast cancer and 13 normal samples), GSE45827 (130 breast cancer and 11 normal samples), and GSE42568 (104 breast cancer and 17 normal samples). Differential gene expression analysis was conducted on each dataset using limma R package (version 4.3). We selected differentially expressed genes (DEGs) based on the following criteria: log2 fold change (log2FC) ≥ 1.0 and *p*-value ≤ 0.05. Concurrently, a compendium of immune-related genes (IRGs) was sourced from the ImmPort database. Intersection analysis via Venn diagram identified 40 overlapping genes common to both the DEG sets and the IRG list. Prognostic analysis of these 40 candidate genes revealed TNFRSF12A as possessing significant prognostic value, designating it as the primary target for subsequent mechanistic investigation (Figure 1).

TNFRSF12A (officially designated Fn14), a fibroblast growth factor-responsive immediate-early gene product, functions as a constitutively active type I transmembrane receptor within the tumor necrosis factor receptor superfamily (TNFRSF) [25]. Current evidence substantiates that TNFRSF12A upregulation promotes bone metastasis in prostate cancer [26] and non-small cell lung carcinoma (NSCLC) [27]. In cutaneous squamous cell carcinoma, TNFRSF12A activation drives neoplastic proliferation [28], while cholangiocarcinoma studies demonstrate its pro-tumorigenic role through tumor microenvironment (TME) remodeling [29]. Collectively, TNFRSF12A propels pan-cancer progression by orchestrating proliferation, metastasis, and TME reprogramming. Therapeutically, leveraging TNFRSF12A’s tumor-selective membrane localization, Li et al. [30] engineered TNFRSF12A × CD3 bispecific T-cell engagers (BiTEs) and TNFRSF12A-targeted CAR-T, both exhibiting potent oncosuppressive efficacy in preclinical models—highlighting substantial translational potential. Consequently, systematic elucidation of TNFRSF12A’s molecular mechanisms and therapeutic vulnerabilities in TNBC will streamline its clinical translation pipeline.

### 3.2. Clinical Validation Confirms Marked TNFRSF12A Upregulation in TNBC Correlating Significantly with Adverse Patient Outcomes

**Figure 2:**
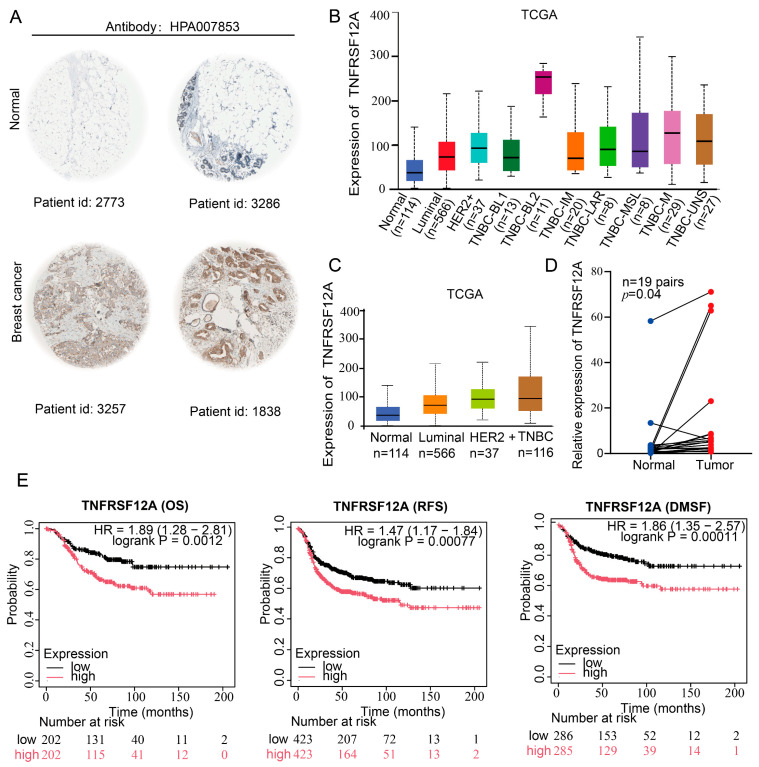
Expression Profile and Prognostic Significance of TNFRSF12A in TNBC. (**A**) Immunohistochemical quantification of TNFRSF12A protein expression in breast carcinoma specimens. (**B**,**C**) Differential mRNA expression patterns of TNFRSF12A across breast adenocarcinoma subtypes and molecular classifications. (The corresponding statistical information is displayed in Tables S1 and S2) (**D**) Significantly elevated TNFRSF12A transcript levels in 19 TNBC tissues versus matched adjacent non-tumor controls (*p* = 0.04). (**E**) Kaplan–Meier survival curves demonstrating adverse prognostic impact of high TNFRSF12A expression (stratified by median cut-off): Overall Survival (OS): HR = 1.89, *p* = 0.0012; Recurrence-Free Survival (RFS): HR = 1.47, *p* = 0.00077; Distant Metastasis-Free Survival (DMFS): HR = 1.86, *p* = 0.00011.

Public omics database analyses revealed TNBC-specific TNFRSF12A overexpression (Figure 2A–C). To further characterize its expression pattern, qRT-PCR profiling demonstrated significantly elevated TNFRSF12A levels in 19 TNBC specimens compared to matched adjacent normal tissues (*p* = 0.04) (Figure 2D). The GSE62931 dataset was analysed, with the result that TNFRSF12A expression levels were found to be significantly higher in triple-negative breast cancer (TNBC) than in non-triple-negative breast cancers (including Her2+, luminal A, and luminal B subtypes) (Figure S1). Further validation through the KM-Plotter prognostic platform (https://kmplot.com/analysis/ (accessed on 22 October 2025)) established that TNFRSF12A overexpression portends poorer TNBC prognosis (Figure 2E), evidenced by the following: shortened overall survival (OS: HR = 1.89, (1.28–2.81); *p* = 0.0012); reduced recurrence-free survival (RFS: HR = 1.47, (1.17–1.84); *p* = 0.00077); worsened distant metastasis-free survival (DMFS: HR = 1.86, (1.35–2.57); *p* = 0.00011). These findings position TNFRSF12A as a promising therapeutic target with clinical translatability in TNBC, underscoring the imperative to dissect its molecular mechanisms for improving patient prognosis.

### 3.3. TNFRSF12A Promotes Angiogenesis in TNBC

**Figure 3:**
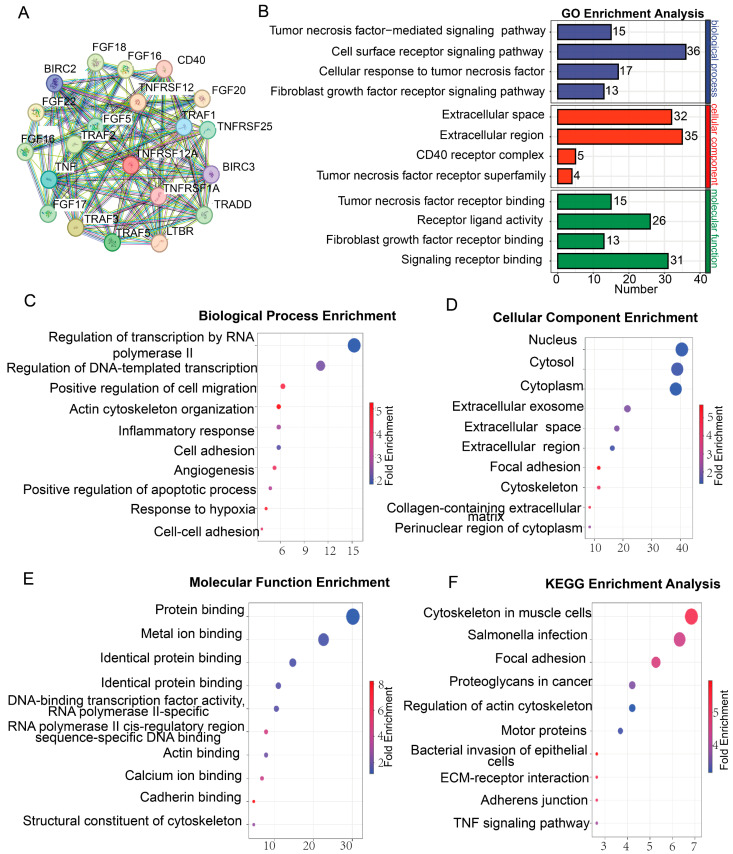
Functional Enrichment Profiling of TNFRSF12A-Associated Pathways. (**A**) PPI network of putative TNFRSF12A-binding partners. (**B**) GO enrichment analysis of potential TNFRSF12A interactors. (**C**) Biological Processes enrichment of TNFRSF12A expression-correlated genes. (**D**) Cellular Components enrichment of TNFRSF12A expression-correlated genes. (**E**) Molecular Functions enrichment of TNFRSF12A expression-correlated genes. (**F**) KEGG Pathways of TNFRSF12A expression-correlated genes.

To delineate the tumor-promoting mechanisms of TNFRSF12A in TNBC, this study used the STRING database to identify proteins that interact with TNFRSF12A. Then, it performed a functional enrichment analysis on these potential interacting proteins. Results demonstrated significant enrichment of these interacting proteins in tumor necrosis factor-mediated signaling pathways, signal transduction cascades, and fibroblast growth factor receptor (FGFR) signaling pathways (Figure 3A,B). This implicates TNFRSF12A in orchestrating TNBC progression through inflammatory microenvironment modulation and growth factor signaling. These findings align with established literature: Aberrant activation of TNF signaling drives tumor cell survival and metastasis via NF-κB-mediated transcriptional reprogramming [31,32], while FGFR signaling underpins the aggressive phenotype of TNBC [33,34,35]. Expression-correlated genes of TNFRSF12A were retrieved from UALCAN platform and subjected to functional enrichment analyses via the DAVID. The top ten enriched terms are presented below: Biological Processes: Preferential enrichment in RNA polymerase II-regulated transcription, positive regulation of cell migration, inflammatory response, angiogenesis, positive regulation of apoptosis, response to hypoxia, and cell–cell adhesion (Figure 3C). Cellular Components: Significant clustering in extracellular regions including exosomes, extracellular matrix, and plasma membrane (Figure 3D). Molecular Functions: Dominant enrichment in metal ion binding, calcium ion binding, protein binding, and RNA polymerase II binding (Figure 3E). KEGG Pathways: Primary enrichment in ECM-receptor interaction and TNF signaling pathway (Figure 3F). Convergence of biological processes—particularly positive regulation of cell migration, angiogenesis, and hypoxia response—suggests involvement in metastatic dissemination and stromal remodeling in TNBC. Critically, exosome and extracellular matrix enrichment in cellular components implies TNFRSF12A may facilitate tumor-stromal crosstalk through exosome-mediated intercellular communication or ECM modification, a mechanism extensively implicated in TNBC distant metastasis. These findings posit TNFRSF12A as a potential orchestrator of pro-angiogenic and pro-metastatic signaling via exosomes. Further mechanistic dissection and validation could position TNFRSF12A as an actionable therapeutic target for TNBC intervention.

### 3.4. TNFRSF12A Knockdown Attenuates Angiogenic Capacity

**Figure 4:**
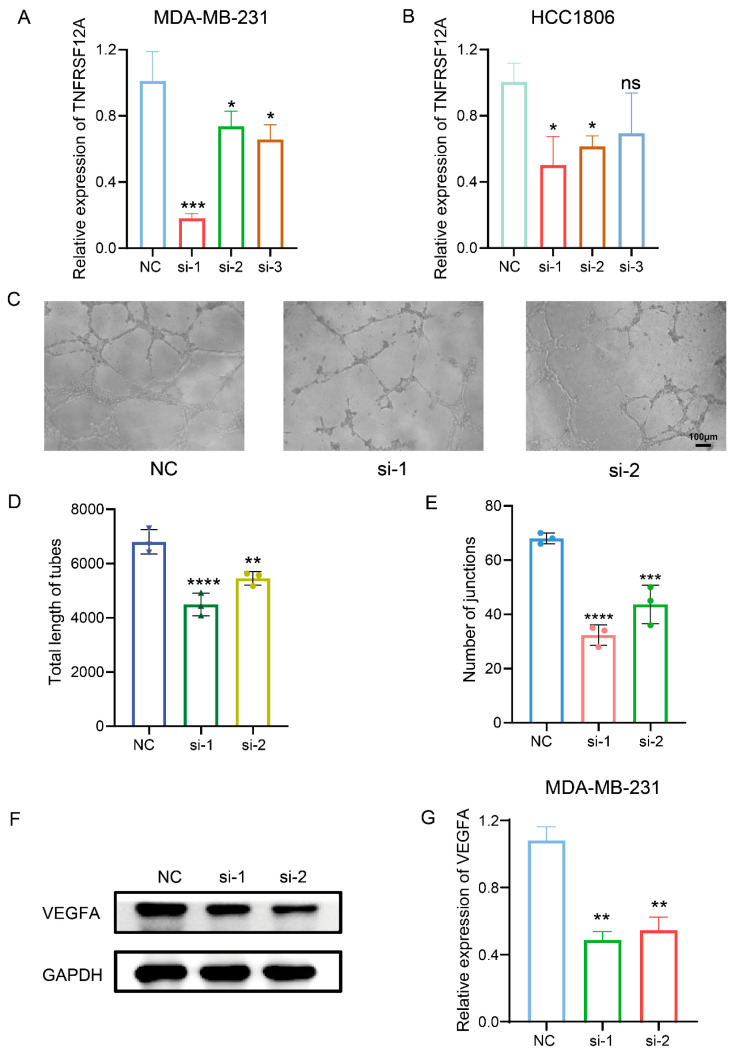
In Vitro Angiogenesis Assay. (**A**,**B**) Validation of siRNA-mediated knockdown efficiency in MDA-MB-231 and HCC1806 cell lines. (**C**) Representative images of HUVEC tubulogenesis. (**D**) Statistical quantification of total tubular length. (**E**) Statistical quantification of branching points. (**F**) Western blot detection of levels of VEGFA protein expression. (**G**) RNA expression levels of VEGFA. *p* < 0.05 (*), *p* < 0.01 (**), *p* < 0.001 (***), *p* < 0.0001 (****), ‘ns’ indicates no statistical significance.

Functional enrichment analyses implicated TNFRSF12A in angiogenic regulation. To validate this hypothesis, we conducted in vitro tubulogenesis assays using HUVECs. Initial validation of siRNA-mediated knockdown efficiency in MDA-MB-231 and HCC1806 cell lines confirmed potent silencing by siRNA-1 and siRNA-2 (Figure 4A,B), which were subsequently employed in functional studies. Tubulogenesis assays demonstrated markedly attenuated vascular network formation upon TNFRSF12A knockdown (Figure 4C–E). To investigate the function of TNFRSF12A, we analyzed the expression of VEGFA—a key factor in angiogenesis—after silencing TNFRSF12A. The experimental results showed that silencing TNFRSF12A simultaneously downregulates the protein and mRNA expression levels of VEGFA (Figure 4F,G). These findings collectively substantiate TNFRSF12A’s role in modulating tumor-associated angiogenesis.

### 3.5. TNFRSF12A Exerts an Inhibitory Impact on Anti-Tumor Immunity by Hindering Immune Cell Infiltration into Tumors and Suppressing T-Cell Cytotoxic Activity

**Figure 5:**
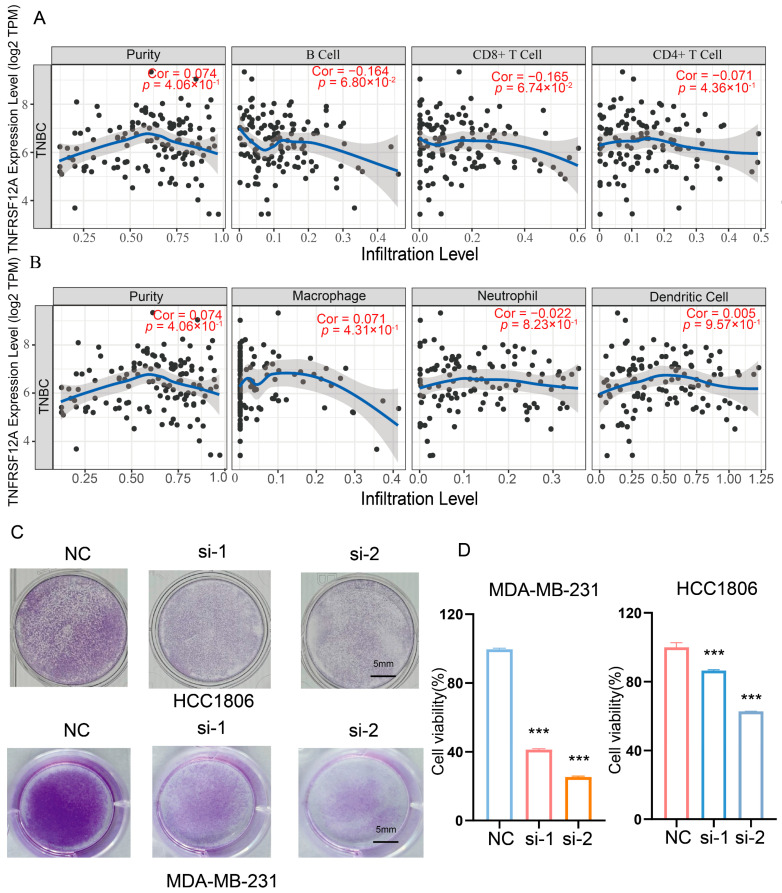
Modulation of the tumor immune landscape by TNFRSF12A in TNBC. (**A**) Correlational profiling of TNFRSF12A expression with infiltration levels of B cell (Cor = −0.164, *p* = 6.8 × 10^−2^), CD8+ T cells (Cor = −0.165, *p* = 6.74 × 10^−2^), and CD4^+^ T cells (Cor = −0.071, *p* = 4.36 × 10^−1^). (**B**) Correlation analysis of TNFRSF12A expression with macrophage (Cor = 0.071, *p* = 4.33 × 10^−1^), neutrophil (Cor = −0.022, *p* = 8.23 × 10^−1^), and dendritic cell infiltration (Cor = −0.005, *p* = 9.57 × 10^−1^). (**C**) Representative data from in vitro T-cell cytotoxicity assays. (**D**) Statistical quantification of T-cell-mediated tumor cell killing. *p* < 0.001 (***).

Tumor immune microenvironment (TIME) constitutes a dynamic ecosystem of immune/stromal cells, cytokines, and metabolites [36], driving oncogenesis through the immunoediting cascade (elimination-equilibrium-escape) [37,38]. Prior studies indicate TNFRSF12A targeting enhances T-cell activation/proliferation in gastric cancer [39]. To elucidate TNFRSF12A’s immunomodulatory role, we first analyzed its correlation with immune infiltration via TIMER (https://cistrome.shinyapps.io/timer/ (accessed on 22 October 2025)), revealing an inverse trend was found with B-cell (Cor = −0.164, *p* = 6.8 × 10^−2^) and CD8^+^ T-cell infiltration (Cor = −0.165, *p* = 6.74 × 10^−3^), alongside variable impacts on other populations: CD4^+^ T cells: Cor = −0.071, *p* = 4.36 × 10^−1^; Macrophages: Cor = 0.071, *p* = 4.33 × 10^−1^; Neutrophils: Cor = −0.022, *p* = 8.23 × 10^−1^; Dendritic cells: Cor = −0.005, *p* = 9.57 × 10^−1^ (Figure 5A,B). In vitro T-cell cytotoxicity assays demonstrated significantly enhanced tumor cell killing upon TNFRSF12A silencing (Figure 5C,D), corroborating bioinformatic data and indicating TNFRSF12A suppresses cytotoxic T-lymphocyte recruitment/effector functions. Mechanistically, TNFRSF12A likely promotes an immune-excluded phenotype—trapping lymphocytes in peri-tumoral stroma rather than infiltrating tumor cores. This spatial constraint impairs immunosurveillance, representing a key immunoediting escape mechanism. Future studies should validate these mechanisms in vivo and delineate downstream lymphocyte-trafficking signals. Our findings provide compelling rationale for targeting TNFRSF12A to reverse immune exclusion, potentially synergizing with checkpoint blockade in “cold” TNBC tumors to improve patient outcomes.

### 3.6. CAF-Specific TNFRSF12A Elevation Correlates with Increased Stromal Abundance

**Figure 6:**
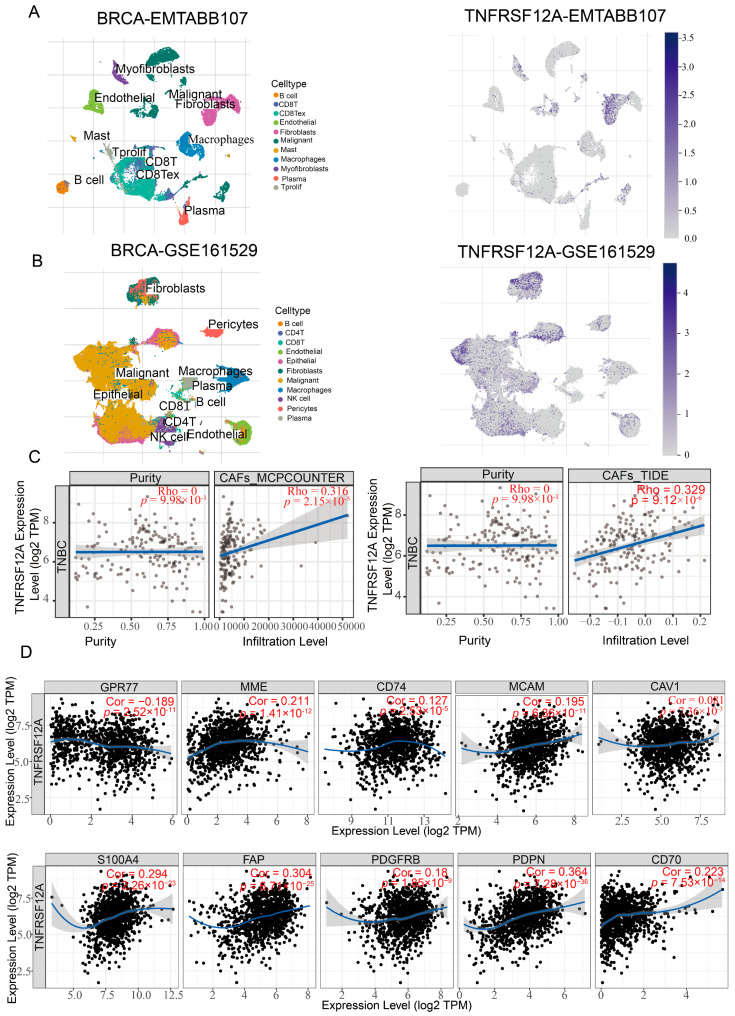
Single-cell expression landscape of TNFRSF12A and its correlation with fibroblast infiltration. (**A**) Expression signatures with single-cell granularity of TNFRSF12A in the BRCA-EMTAB11077 dataset. (**B**) Expression signatures with single-cell granularity of TNFRSF12A in the BRCA-GSE161529 dataset. (**C**) Correlation analysis between TNFRSF12A expression levels and fibroblast infiltration levels (MCPcounter algorithm:ρ = 0.316, *p* = 2.15 × 10^−5^; TIDE algorithm: ρ = 0.329, *p* = 9.12 × 10^−6^). (**D**) Correlation analysis between TNFRSF12A and fibroblast signature genes (GPR77 (Cor = −0.189, *p* = 2.52 × 10^−10^; MME (Cor = 0.221, *p* = 1.41 × 10^−12^; CD74 (Cor = 0.127, *p* = 2,52 × 10^−5^); MCAM (Cor = 0.195, *p* = 6.36 × 10^−11^); CAV1 (Cor = 0.081, *p* = 7.36 × 10^−3^); S100A4 (Cor = 0.294, *p* = 2.26 × 10^−23^); FAP (Cor = 0.304, *p* = 6.21 × 10^−25^); PDGFRB (Cor = 0.18, *p* = 1.85 × 10^−9^); PDPN (Cor = 0.364, *p* = 7.28 × 10^−36^); CD70 (Cor = 0.223, *p* = 7.53 × 10^−14^).

The tumor microenvironment (TME)—a complex ecosystem comprising malignant cells, immune populations, stromal constituents, and extracellular matrix—plays a pivotal role in tumorigenesis, metastasis, and therapeutic resistance [40,41,42,43]. Advancements in single-cell genomics now enable unprecedented resolution for deconvoluting TME cellular architecture, state transitions, and molecular networks [44]. To delineate TNFRSF12A’s functional role within the TME, we leveraged the TISCH2 database (http://tisch.comp-genomics.org (accessed on 22 October 2025)) to map its expression landscape across cellular subtypes in breast carcinoma. Our analysis revealed predominant TNFRSF12A enrichment in cancer-associated fibroblasts (CAFs) (Figure 6A,B). Subsequent analyses revealed a direct relationship with increased CAF abundance in TNBC (MCPcounter algorithm: ρ = 0.316, *p* = 2.15 × 10^−5^; TIDE algorithm: ρ = 0.329, *p* = 9.12 × 10^−6^) (Figure 6C,D). Moreover, correlational analysis demonstrated pervasive positive associations between TNFRSF12A and established CAF marker genes (GPR77 (cor = −0.189, *p* = 2.52 × 10^−10^); MME (Cor = 0.221, *p* = 1.41 × 10^−12^); CD74 (Cor = 0.127, *p* = 2.52 × 10^−5^); MCAM (cor = 0.195, *p* = 6.36 × 10^−11^); CAV1 (Cor = 0.081, *p* = 7.36 × 10^−3^); S100A4 (Cor = 0.294, *p* = 2.26 × 10^−23^); FAP (Cor = 0.304, *p* = 6.21 × 10^−25^); PDGFRB (Cor = 0.18, *p* = 1.85 × 10^−9^); PDPN (Cor = 0.364, *p* = 7.28 × 10^−36^); CD70 (Cor = 0.223, *p* = 7.53 × 10^−14^)) (Figure 6). Cancer-associated fibroblasts (CAFs) constitute pivotal stromal components within the tumor microenvironment (TME) that drive malignant progression [45]. Expression of their heterogeneity markers (e.g., FAP [46], PDGFRB [47], S100A4 [48,49]) correlates significantly with breast cancer invasiveness and adverse prognosis. Our study identified CAF-specific TNFRSF12A overexpression, aligning with prior observations: in hepatocellular carcinoma, elevated TNFRSF12A similarly associates with CAF enrichment and Treg infiltration, accelerating tumor aggressiveness via apoptotic and invasive pathway dysregulation [50,51]. The pervasive positive correlation between TNFRSF12A and key CAF markers—particularly its strong association with FAP (ρ = 0.304) and PDPN (*p* = 0.364)—implies its involvement in TME remodeling through dual mechanisms: FAP^+^ CAFs secrete matrix metalloproteinases (MMPs) that degrade extracellular matrix [46], facilitating peritumoral dissemination; PDPN^+^ CAFs exclude T cells via CXCL12-mediated chemorepulsion [52,53], establishing immune-privileged niches. Notably, TNFRSF12A-PDPN co-expression (ρ = 0.364) suggests synergistic suppression of anti-tumor immunity. However, this study lacks direct experimental validation of TNFRSF12A expression in CAFs. Further research is needed in this area. Consequently, future investigations should delineate TNFRSF12A’s role in CAF subset differentiation and validate its therapeutic targetability or prognostic utility, providing critical rationale for novel TNBC treatment strategies.

### 3.7. Elevated TNFRSF12A Expression Promotes Sensitivity to Docetaxel Treatment

**Figure 7:**
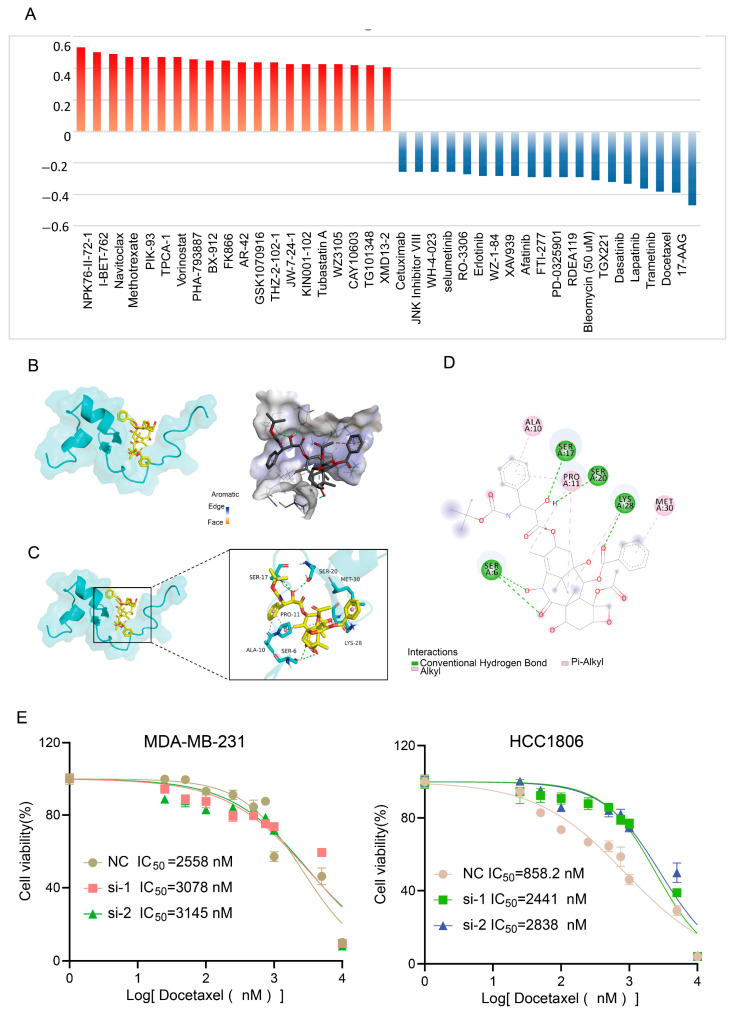
Drug resistance analysis of TNFRSF12A. (**A**) Correlation analysis between TNFRSF12A expression levels and therapeutic drug resistance. (**B**) Three-dimensional structural representations of TNFRSF12A and the drug docetaxel. (**C**) Three-dimensional molecular docking model depicting the interaction between TNFRSF12A and docetaxel. (**D**) Two-dimensional interaction diagram of the molecular docking results between TNFRSF12A and docetaxel. (**E**) Determination of IC_50_ values for docetaxel in TNBC cell lines treated with varying concentrations of the drug, with absorbance measured at 450 nm to assess cell viability.

The triple-negative phenotype (lacking ER, PR, and HER2) drives therapeutic limitations, elevated resistance risk, and inferior survival outcomes [26,27]. This study focuses on TNFRSF12A, which is highly expressed in TNBC, investigating its potential role in regulating tumor drug resistance. Drug resistance analysis utilizing the GSCA database integrated with the GDSC drug database revealed that elevated TNFRSF12A expression levels correlated with increased sensitivity to docetaxel (Figure 7A). To elucidate the underlying molecular mechanism, molecular docking analysis (Figure 7B–D) identified a potential interaction between TNFRSF12A and docetaxel (binding energy: −6.7 kcal/mol). The docking model indicates that residues 10Ala, 11Pro, 28Lys, and 30Met of TNFRSF12A form hydrophobic interactions with docetaxel, while residues 17Ser, 20Ser, 28Lys, and 6Ser form hydrogen bonds. Furthermore, in vitro CCK-8 assay results confirmed that knockdown of TNFRSF12A led to increased IC_50_ values for docetaxel in both MDA-MB-231 and HCC1806 cells (Figure 7E). These results suggest that TNFRSF12A could be used as a biomarker to predict docetaxel efficacy and as a target to enhance the effectiveness of drugs. However, further in vitro and in vivo experimental studies are required to validate these functions and understand the underlying mechanisms. Consequently, combining TNFRSF12A-targeted therapy with docetaxel chemotherapy represents a potential strategy for treating TNBC patients. The key advantage of such combination therapy lies in generating synergistic effects through drugs with complementary mechanisms. During tumor treatment, secondary genetic mutations or bypass pathway activation often led to monotherapy failure; combination therapy significantly delays disease progression by concurrently inhibiting multiple resistance pathways. However, realizing this combination approach requires further elucidation of the precise mechanism of action of TNFRSF12A in TNBC.

## 4. Conclusions

This study systematically elucidated the pro-tumor mechanism and therapeutic value of TNFRSF12A in triple-negative breast cancer (TNBC) by integrating multi-omics analysis with experimental validation. Using public databases, the study identified TNFRSF12A overexpression in TNBC tissues, which was confirmed in clinical TNBC patient samples. Additionally, Prognostic analysis indicated that high TNFRSF12A expression is significantly linked to poorer overall survival (OS, HR = 1.89), recurrence-free survival (RFS, HR = 1.47), and distant metastasis-free survival (DMFS, HR = 1.86). These findings support the potential of TNFRSF12A as an independent prognostic marker for TNBC. Subsequent functional enrichment analysis indicated that TNFRSF12A was markedly enriched in positive regulation of cell migration, angiogenesis, and hypoxia response, thereby suggesting its potential involvement in the metastatic spread and microenvironment remodeling of TNBC. In vitro experiments demonstrated that silencing TNFRSF12A significantly inhibited the tube-forming ability of HUVEC cells (*p* < 0.01), corroborating the activation of the angiogenesis pathway suggested by functional enrichment analysis. Bioinformatics analysis revealed a negative correlation between TNFRSF12A and CD8^+^ T cell (Cor = −0.165) and B cell (Cor = −0.164) infiltration. In vitro experiments demonstrated that silencing TNFRSF12A enhances T cell killing efficiency (*p* < 0.05). The fifth point indicates that it weakens antitumor immunity by inducing an “immune rejection” phenotype. A single-cell transcriptomics analysis (TISCH2 database) revealed that TNFRSF12A is highly expressed in cancer-associated fibroblasts (CAFs) and positively correlated with CAF markers (FAP: *ρ* = 0.304; PDPN: *ρ* = 0.364). Quantitative analysis confirmed parallel upregulation of PDPN and CAF infiltration markers in TNBC biopsies. This correlation was statistically significant. The results of this study suggest that the progression of TNBC is promoted by CAF-mediated microenvironmental remodeling. A recent study revealed that high TNFRSF12A expression enhances docetaxel sensitivity. Moreover, molecular docking revealed that the drug directly binds to the protein via key residues (10ALA/28LYS, etc.) with a binding energy of −6.7 kcal/mol. Gene silencing experiments confirmed that TNFRSF12A deficiency increases the docetaxel IC_50_ value in MDA-MB-231/HCC1806 cells, supporting its role as a chemotherapy sensitization target. Consequently, a synergistic treatment regimen targeting TNFRSF12A in combination with docetaxel is proposed to overcome TNBC resistance by reversing immune suppression and enhancing chemotherapy response. This study is pioneering in its exploration of the multifaceted role of TNFRSF12A in TNBC, delineating its function as a “diagnostic marker–microenvironment regulatory factor–chemotherapy sensitization target”. The findings of this study lay the groundwork for the development of combination therapies that capitalize on the potential of TNFRSF12A inhibitors.

## Figures and Tables

**Table 1:** Sequence of TNFRSF12A siRNA.

siRNA Name	Sequence
siRNA-1	5′-AGAGAGAAGTTCACCACC-3′
siRNA-2	5′-CACTCATCATTCATTCATC-3′

**Table 2:** Primer sequence for qPCR.

Gene Name	Forward 5′--3′	Reverse 5′--3′
TNFRSF12A	GACCTGGACAAGTGCAT	GGTGGTGAACTTCTCTCTC
U6	CTCGCTTCGGCAGCACA	AACGCTTCACGAATTTGCGT
β-actin	CTCTTCCAGCCTTCCTTCCT	AGCACTGTGTTGGCGTACAG
VEGFA	AACTTTCTGCTGTCTTGG	ACTTCGTGATGATTCTGC

## Data Availability

All datasets analyzed in this study are available for download from the T ImmPort database (https://immport.org/) and the GEO database (https://www.ncbi.nlm.nih.gov/geo/).

## Supplementary Materials

The following supporting information can be downloaded at: https://www.mdpi.com/article/10.3390/biology14111513/s1, Figure S1: Analysis of TNFRSF12A expression levels in TNBC and non-TNBC (Her2+ type, Luminal A type, Luminal B type) in the dataset GSE62931. Table S1: Statistical significance in the comparison of major molecular subtypes (with TNBC) of Triple-negative breast cancer. Table S2: Statistical significance in the comparison of major molecular subtypes of Triple-negative breast cancer.

## Abbreviations

The following abbreviations are used in this manuscript:

Abbreviations	Full Name
TNBC	Triple-negative breast cancer
TCGA	The Cancer Genome Atlas
GEO	Gene Expression Omnibus
OS	Overall survival
RFS	Recurrence-Free Survival
DMFS	Distant Metastasis Free Survival
HR	Hazard Ratio
CAFs	Cancer-associated fibroblasts
GO	Gene Ontology
KEGG	Kyoto Encyclopedia of Genes and Genomes
ECM	Extracellular matrix
HUVECs	Human Umbilical Vein Endothelial Cells
DEGs	Differentially expressed genes
PPI	Protein–Protein Interaction Networks
BP	Biological process
MF	Molecular function
CC	Cellular component
FGF	Fibroblast growth factor
FGFR	Fibroblast growth factor receptor
Cor	Correlation coefficient
