# Supplementary material for: Over-Expression of TNFRSF12A Promotes Immune Suppression and Facilitates Angiogenesis in Triple-Negative Breast Cancer
Source: Biology (Basel). 2025 Oct 28;14(11):1513. doi: 10.3390/biology14111513 (PMC12650428; doi:10.3390/biology14111513)
Supplement: Supplementary file 1 [file biology-14-01513-s001.zip › biology-3850235-supplementary.pdf]

Over-Expression of TNFRSF12A Promotes Immune Suppression and Facilitates Angiogenesis in Triple-Negative Breast Cancer

Can Jiang, Zhengwei Zhou, Guang Shu, Gang Yin and Maonan Wang *

Department of Pathology, Xiangya School of Basic Medical Sciences, Central South University, Changsha 410013, China
* Correspondence: maonanwang@csu.edu.cn

Supplementary Figures

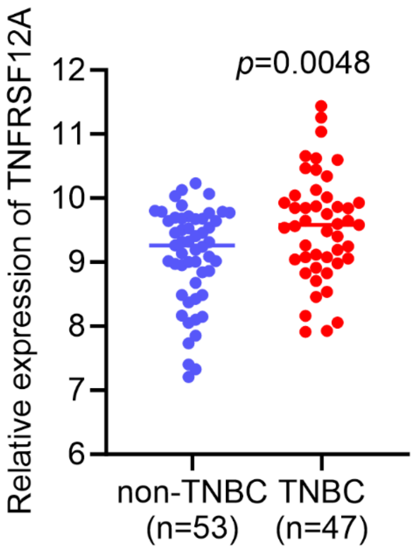

Figure S1. Analysis of TNFRSF12A expression levels in TNBC and non-TNBC (Her2+ type, Luminal A type, Luminal B type) in the dataset GSE62931.

Supplementary Tables

Table S1. Statistical significance in the comparison of major molecular subtypes (with TNBC) of Triple-negative breast cancer

Comparison	Statistical significance
Normal-vs-Luminal	1.50990331349021E-14
Normal-vs-HER2Pos	1.57580000004209E-06
Normal-vs-TNBC-BL1	4.359800E-03
Normal-vs-TNBC-BL2	5.003900E-04
Normal-vs-TNBC-IM	7.218600E-03
Normal-vs-TNBC-LAR	6.361600E-02
Normal-vs-TNBC-MSL	8.284100E-02
Normal-vs-TNBC-M	6.17290000000104E-05

Normal-vs-TNBC-UNS	4.37460000000112E-05
Luminal-vs-HER2Pos	1.194610E-01
Luminal-vs-TNBC-BL1	8.825600E-01
Luminal-vs-TNBC-BL2	1.804440E-03
Luminal-vs-TNBC-IM	7.943800E-01
Luminal-vs-TNBC-LAR	5.690000E-01
Luminal-vs-TNBC-MSL	3.402600E-01
Luminal-vs-TNBC-M	3.324500E-02
Luminal-vs-TNBC-UNS	9.030400E-02
HER2Pos-vs-TNBC-BL1	2.889600E-01
HER2Pos-vs-TNBC-BL2	3.756800E-03
HER2Pos-vs-TNBC-IM	4.361600E-01
HER2Pos-vs-TNBC-LAR	8.698800E-01
HER2Pos-vs-TNBC-MSL	5.885400E-01
HER2Pos-vs-TNBC-M	3.048200E-01
HER2Pos-vs-TNBC-UNS	7.702200E-01

Table S2. Statistical significance in the comparison of major molecular subtypes of Triple-negative breast cancer

Comparison	Statistical significance
Normal-vs-Luminal	1.50990331349021E-14
Normal-vs-HER2 Positive	1.57580000004209E-06
Normal-vs-TNBC	7.79043496379472E-13
Luminal-vs-HER2 Positive	1.194610E-01
Luminal-vs-TNBC	1.644440E-04
HER2 Positive-vs-TNBC	1.544760E-01
